# The Synergistic Effect of Temperature and Loading Rate on Deformation for Thermoplastic Fiber Metal Laminates

**DOI:** 10.3390/ma14154210

**Published:** 2021-07-28

**Authors:** Kai Jin, Shanyong Xuan, Jie Tao, Yujie Chen

**Affiliations:** 1 College of Material Science and Engineering, Ocean University of China, Qingdao 266100, China; jinkai@ouc.edu.cn; 2 State-Owned Wuhu Machinery Factory, Wuhu 241000, China; 3 College of Material Science and Technology, Nanjing University of Aeronautics and Astronautics, Nanjing 211106, China; chenyujie@nuaa.edu.cn

**Keywords:** Al/Gf/PP laminate, deformation, temperature, loading speed, formability

## Abstract

The glass fiber reinforced polypropylene/AA2024 hybrid laminates (short for Al/Gf/PP laminates) as structural materials were prepared and formed by hot pressing. The synergistic effects of temperature and loading speed on the laminate deformation under tensile and bending conditions were investigated and analyzed in this study. In tension, stress–strain curves presented bimodal types effected by tensile rates and temperatures. The state of PP resin determines the mechanical behavior of the FMLs. The tensile rate has no effect on FML deformation without heating or over the melting point of PP resin (about 170 °C). The softening point of PP resin (about 100 °C) is characteristic temperature. When the temperature exceeds the softening point but does not reach the melting point, the tensile strength and elongation will demonstrate coordinated growth at a relatively high tensile speed. The efficiency of fiber bridging is affected significantly since the resin is the medium that transfers load from the metal to the fiber. Under bending, the curves presented a waterfall decrement with temperature increment. The softening point of resin matrix is the key in a bending process. When the temperature is near the softening point, deformation is sensitive to both the temperature and the loading speed to a certain extent. If temperature is lower than softening point, deformation is mainly guided by temperature. If the temperature is beyond the softening point, loading speed is in a leading position of deformation. The bending strength gradually increases with loading rate. By using these deformation characteristics, the deformation of the thermoplastic laminates can be controlled in stamping or other plastic forming processes for thermoplastic fiber metal laminates.

## 1. Introduction

Fiber metal laminates (FMLs) have developed rapidly in the last two decades as a family of hybrid materials consisting of bonded thin metal sheets and fiber/adhesive layers. This laminated structure provides the material with excellent fatigue, impact, and damage tolerance characteristics and a low density [1,2,3,4,5]. Producing a component with complex geometries typically requires preparing, curing, and forming stages, which are very labor intensive and expensive. In order to introduce FML materials to automotive and rail transit manufacturing industries, a fast and cheap forming method is required.

A thermoforming technique, such as hot stamping or hot pressing, has been used to process glass fiber reinforced polypropylene (PP). A hemispherical mold was used to form parts from pre-heated flat pre-consolidated laminates. Then, pre-consolidated laminates were heated and formed by contact heating above the PP melting temperature in a matched metal tool [6,7,8,9,10]. Bogaerts et al. [11,12,13,14] attributed the forming deformation to forming rate, blank temperature, and blank holder force. Harrison et al. [15] developed an approach to modelling the rheology of composite sheet forming that considered the strain rate effects on inter- and intra-tow stress and polymer viscosities. A study of Mosse et al. [16] presented that the temperature distribution was critical to the formability of the FMLs. A low temperature may result in cracking of the composite and delamination between the layers. Conversely, a high temperature may cause excessive thinning.

Most investigations have only been concerned with the parameter study during a forming process, such as temperature, blank holder force, and punch rate [17,18,19]. However, the dynamic deformation characteristic under different forming conditions and the interplay effect of control factors have not been studied in detail. The stress state of the thermoplastic FMLs during hot forming is constantly changing. In other words, deformation is changed dynamically when the forming area goes through a stress state transition or a temperature state transition. In this study, under tensile and bending conditions, the effect of temperature and loading speed on the deformation of Al/Gf/PP laminate was investigated. In order to indicate the synergistic effect, various temperatures and loading speeds were adopted. This study offer possibility for quantitative analysis and prediction in the deformation process of thermoplastic FMLs during a hot forming process.

## 2. Experiment

### 2.1. Materials

For processing the thermoplastic FMLs, there were several raw materials and devices prepared. The glass fiber reinforced polypropylene (GFRP) prepregs were supplied from the Shanghai Genius Advanced Materials (Group) Co., Ltd. (Shanghai, China) and 2024-T3 aluminum alloy was used for the metal skin of the FMLs, which was supplied by Southwest Aluminum (Group) Co., Ltd. (Chongqing, China).

### 2.2. Sample Preparation

**Figure 1:**
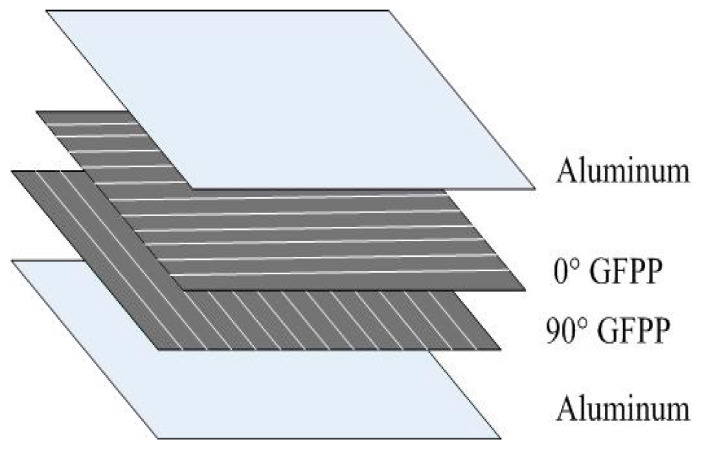
Lay-up of fiber metal laminates.

**Figure 2:**
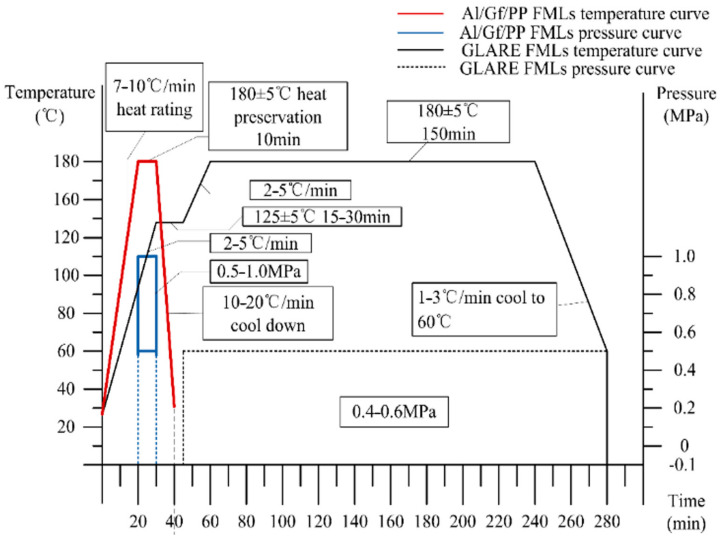
Curing curve of Al/Gf/PP laminate.

The glass fiber reinforced polypropylene FMLs plates was 2/1 lay-up, which was a sandwich structure. Two prepregs were used as a monolithic core of the FMLs, which is named a fiber layer. Here, a typical cross-ply laminated GFRP was used to ensure structural symmetry. The ply sequence was (0°/90°) as shown in Figure 1. Meanwhile, two aluminum sheets were laid on the outside as the skin. The aluminum alloy sheets were cleaned by absolute ethyl alcohol (Beijing Chemical Works, Beijing, China) for degreasing, then treated with a phosphoric acid anodizing process to grow an anodized film. The anodized film on the aluminum surface contained two layers. A porous layer had tubular holes perpendicular to the surface of the aluminum alloy, resulting in greater roughness, while a barrier layer was composed of thin and dense alumina, leading to the characteristics of high strength and electric insulation. The thermoplastic FMLs were bonded through hot pressing in a vulcanizing machine (XLB-D 0.1MN, 400 mm × 400 mm × 1 mm) using a press and temperature processing curves listed in Figure 2.

### 2.3. Mechanical Test

The tensile test was performed according to the standard of ASTM 3039-17. Specimens (200 mm × 20 mm) were prepared by waterjet cutting. The laser measuring instrument (Zwick, Germany) was adopted to measure the deformation precisely. The three-point bending test was implemented according to the standard of ASTM D790-17. Samples were cut into 70 mm × 25 mm (length × width), in which 0° fibers were parallel to the length direction, while 90° fibers were perpendicular to it. The span to thickness ratio (L/H) was set as 16 for the bending test based on previous research result [20]. The temperatures used in these tests were 25.0 °C, 80.0 °C, 140.0 °C, 160.0 °C, and 180.0 °C, while the loading speeds were 1.0 mm/min, 10.0 mm/min, and 100.0 mm/min. For each test condition, five specimens were used. The curve that is at average level in these specimens in each condition was selected as the test result after removing error curves.

## 3. Results and Discussions

### 3.1. Tensile Characteristics

**Figure 3:**
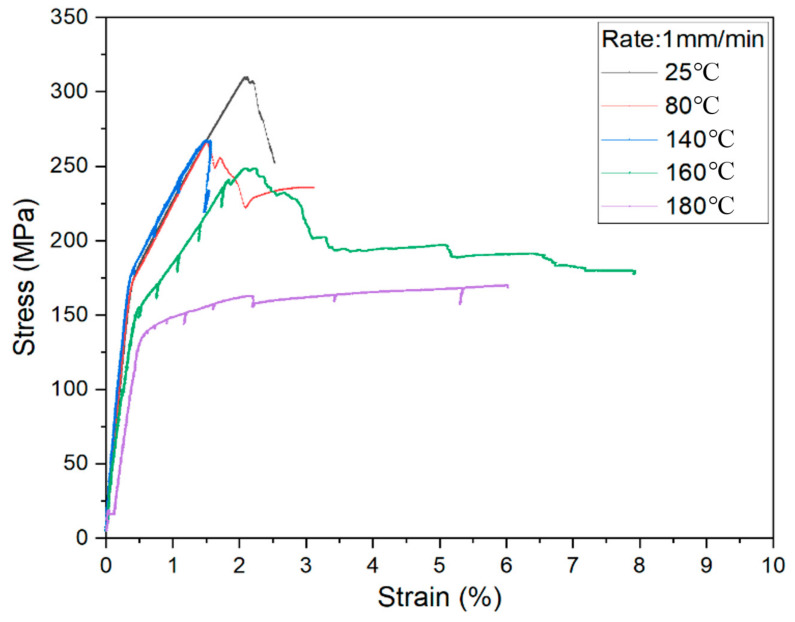
Stress-strain curve under 1 mm/min stretching rate.

The stress–strain curve tested in 1 mm/min stretching rate is shown in Figure 3. There are three different curve types controlled by temperatures. The melting point of PP resin is about 170 °C. The curve at 180 °C is distinct from others because of resin melt. In addition, the softening point of PP resin is about 100 °C. Therefore, the curve without heating is different from other three curves.

**Figure 4:**
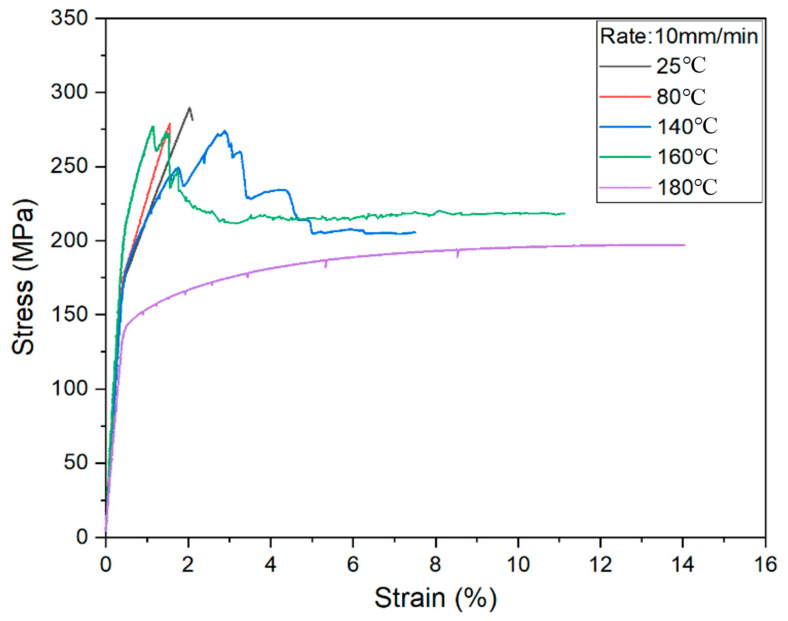
Stress-strain curves under 10 mm/min stretching rate.

If the samples were stretched at 10 mm/min, the stress–strain curve became complete (see Figure 4). It can be seen that the loading rate has an obvious effect on FML deformation. The formability of the material is very limited if the temperature is lower than the softening point of PP resin. Meanwhile, the deformation property bottoms out if the material is heated near the melting temperature. Besides, when the temperature is 180 °C, the stress–strain curve is similar to that under the 1 mm/min stretching rate. This means the loading rate may not affect the FML deformation if the resin matrix is in a viscous state.

**Figure 5:**
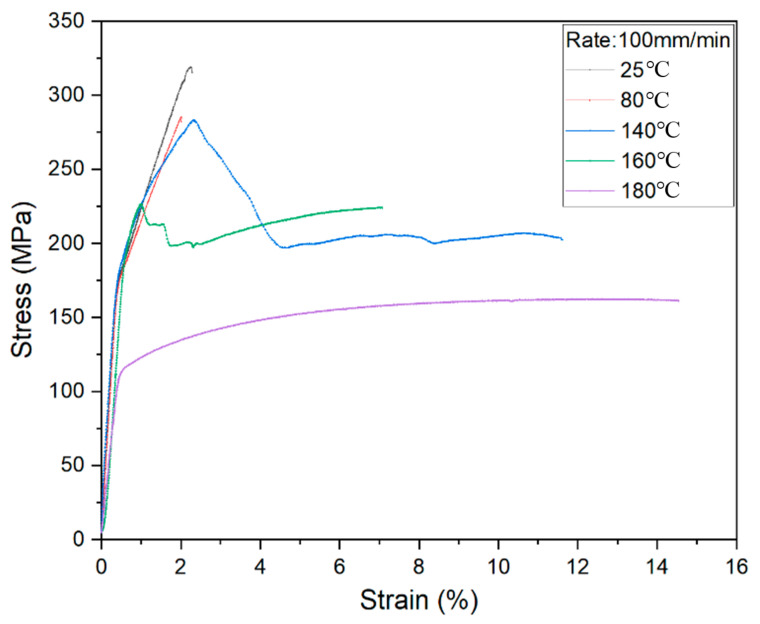
Stress-strain curves under 100 mm/min stretching rate.

The stress–strain curve became the shapes as shown in Figure 5, increasing the stretching rate to 100 mm/min. It can be found that the formability at 160 °C becomes worse than that at the 10 mm/min stretching rate. If the temperature is greater than the PP resin melt point (170 °C), the curve in 100 mm/min is the same as two curves in 1 mm/min and in 10 mm/min. Therefore, the melt point of resin matrix is the key to deformation control.

**Figure 6:**
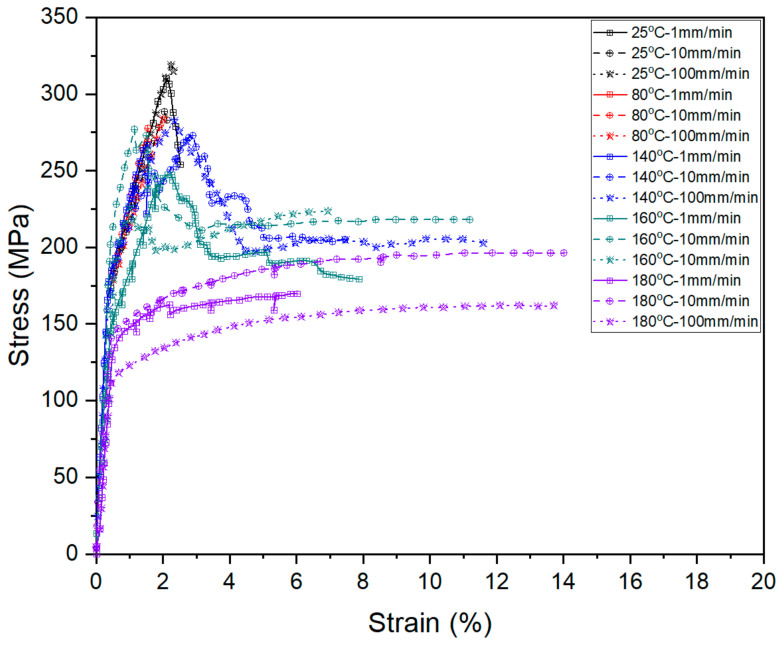
Tensile stress-strain curves under different temperatures and stretching rates.

For further visual comparison, three-dimensional tensile curves (see Figure 6) were used to indicate the synergistic effect of temperature and loading rate. The tensile curves in three tension speeds at room temperature are not much different. The reason for this is that, if adopting a high loading rate, the PP molecular chain cannot change its configuration in a short time through the internal rotation, resulting in no flexibility [21]. It can be concluded that the loading rate has no effect on FML deformation without heating. On the other hand, the shape of three curves at 180 °C is quite similar. Once the temperature is over the melting point of PP resin (about 170 °C), the strength of the material drops sharply. For fiber layers, because PP is completely melted, the resin matrix is in a viscous state. The matrix is almost unable to effectively transfer stress to fibers. Metal layers mainly carry loads of the FMLs at this stage. Therefore, the state of PP resin determines the mechanical behavior of the FMLs.

The softening point of PP resin (about 100 °C) is characteristic temperature. When the temperature exceeds the softening point but does not reach the melting point, the tensile strength and elongation will demonstrate coordinated growth again at a relatively high tensile speed. Since the critical shear stress decreases and the crystal dislocation becomes relatively easy over 100 °C, the plasticity of metal improves obviously. For fiber layers, the state of PP resin is changed to a high-elastic state. According to the research of Guo et al. [22], the resin matrix deforms greatly under shear when the resin softens. The efficiency of fiber bridging is affected significantly since the resin is the medium that transfers load from the metal to the fiber. Meanwhile, the state of PP resin is relatively stable when the temperature is between the softening point and the melting point. Here, the resin has no obvious effect on FMLs. Hence, the FMLs should be formed in this temperature range with a high drawing speed.

When the temperature is close to the melting point of PP resin (170 °C), there are some jagged ripples in the stress–strain curves. For metal layers, dynamic aging strain occurred at that time, which is called the Portevin–Lechatelier (PL) effect [23]. Based on the effect, the air mass generated by diffused atoms impedes dislocation motion [24]. Thus, greater loading is required to generate plastic deformation, resulting in the rise of strength and the existence of jagged ripples. These factors may affect the forming accuracy of parts.

It also can be seen that loading rate and temperature control the deformation of FMLs synergistically. The FMLs demonstrate a rapid increase for the elastic-plastic deformation but a slower increase for the full plastic deformation if adopting 10.0 mm/min around room temperature. When approaching 100.0 °C, a remarkable decrease appears due to reaching the softening point. More specifically, the decrease of curve resulting from state transition. Stress transmission from metal skin to glass fiber through resin has been blocked suddenly. The synergistic effect of temperature and loading rate could be observed more clearly at the region where the temperature is between the softening point and the melting point of PP resin. In addition, it can be seen that the sensitivity of Al/Gf/PP laminates to temperature and loading rate is different. The sensitivity to temperature is higher than loading rate. The influence of temperature is mainly realized by changing the state of PP resin.

**Figure 7:**
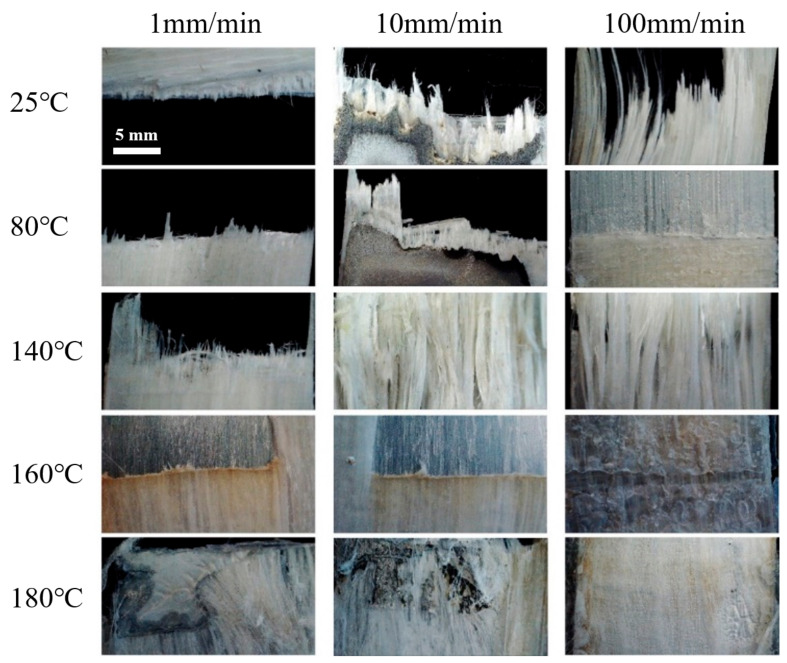
Failure forms of tensile test samples.

The synergistic effect of temperature and loading rate can be further proved by the failure modes of FMLs (see Figure 7). The failure modes can be divided into two types: mode I is fiber breakage (below 160 °C); mode II is fiber slipping (at or over 160 °C). In mode I, the fiber layers always behave elastically, and the metal layers are elastoplastic. The failure is controlled by the fiber failure strain, which means a brittle fracture behavior. In mode II, the temperature reaches the melting point of PP resin. The fiber arrangement is changed with the overflow of melted PP resin, which indicates failure of the bridging effect. The metal layers are still elastoplastic, but the fiber layers cannot bear the external load. Here, the matrix is in a viscous state at this time and there is no failure behavior such as fiber fracture. The failure of FMLs depends on the ductile fracture of metal layers. Therefore, the melt point of PP resin matrix is a characteristic temperature that controls the tensile behavior of Al/Gf/PP laminates.

### 3.2. Bending Characteristics

**Figure 8:**
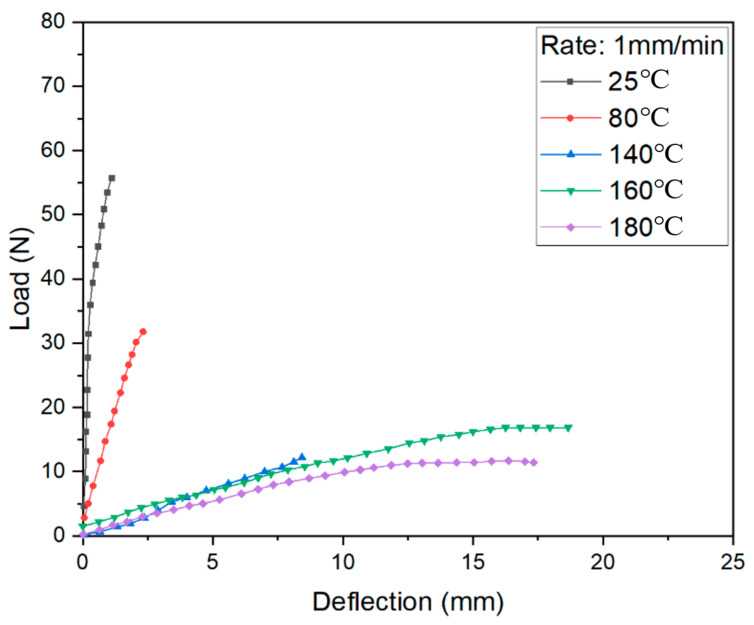
Load-displacement curves and fitting surface under 1 mm/min loading rate.

**Figure 9:**
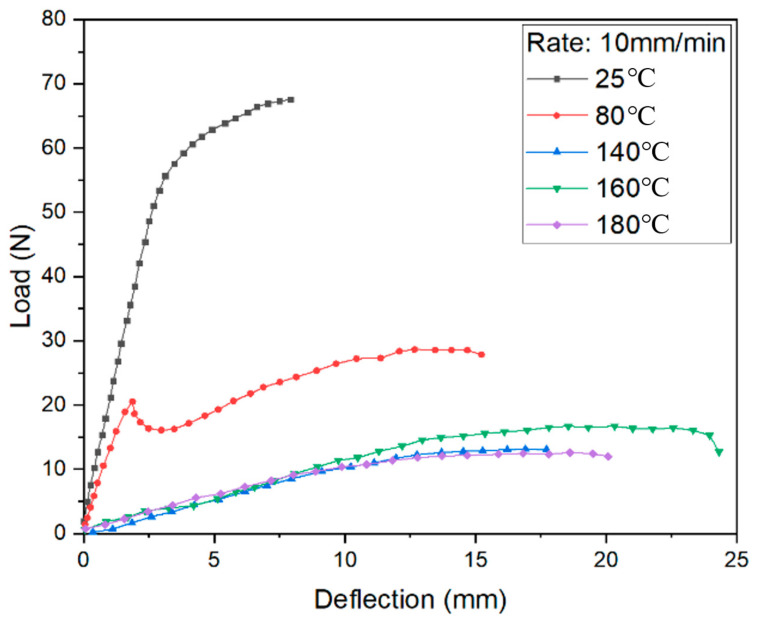
Load-displacement curves and fitting surface under 10 mm/min loading rate.

**Figure 10:**
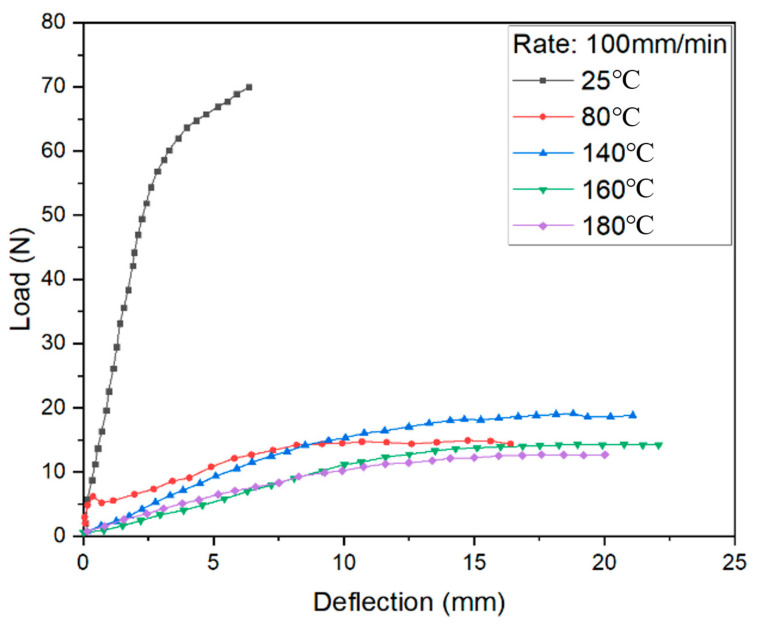
Load-displacement curves and fitting surface under 100 mm/min loading rate.

The bending test results under different load conditions are shown in Figure 8, Figure 9 and Figure 10. The curves at 140, 160, and 180 °C are almost the same as loaded by 1 mm/min and 10 mm/min, with some difference if using 100 mm/min. It can be found that the melt point of resin is obviously no longer key to the control of bending deformation.

Meanwhile, the strength is absolutely high at room temperature. When the temperature is close to the softening point of PP resin, the bending strength decreases greatly. If the temperature exceeds the softening point but does not reach the melting point, the strength is in a stable state while the specific elongation increases. It can be seen that the softening temperature of PP resin is a clear cut-off point. Once the resin softens, the resistance to deformation decreases sharply. The deformation behavior is sensitive to loading speed when the resin is just in an elastic state.

**Figure 11:**
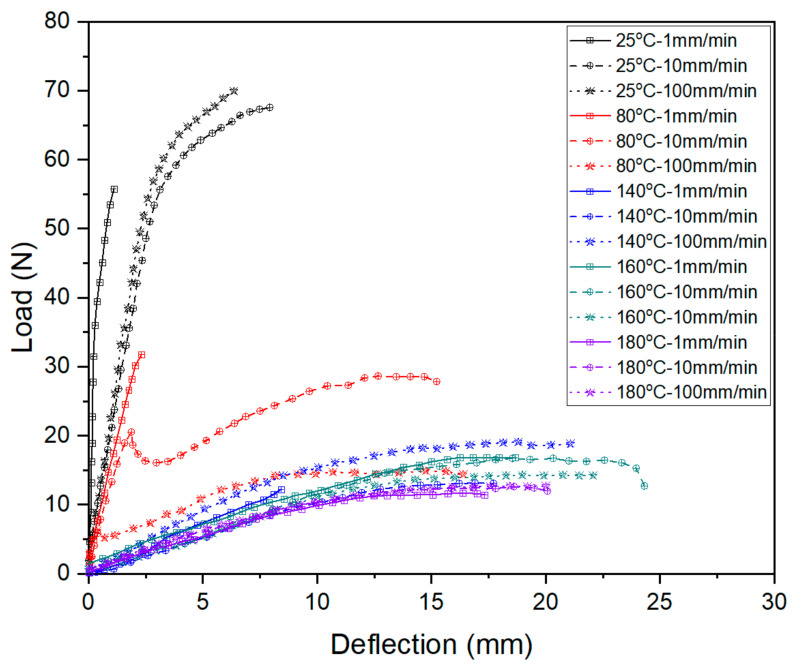
Bending load-deflection curves under different temperatures and speeds.

Comparing the results curves shown in Figure 11, the deformation behaviors could be described in three stages. At the first stage during bending, the pressure head contacted the upper surface of the FMLs, which generated an axial compression. Meanwhile, the lower surface was under axial tension, leading to thickening in the upper metal layers and thinning in lower metal layers. The bending stiffness increases with the increase of loading rate.

If the temperature rises close to softening point of PP resin, the deformation reaches to the second stage. The stiffness decreases significantly, and the elongation increases. From the perspective of metal layers, the upper layer is subjected to the action of compressive stress, which can effectively inhibit the intergranular deformation, at the same time eliminating some micro damage caused by plastic deformation. Moreover, the matrix cannot provide enough support since it reaches an elastic state.

When the temperature is closer to melting point, there is a smooth deformation surface. The resin changes to the viscous state. The matrix can only transmit a part of the stress and bear shear stress. The FMLs can still withstand a bending load by metal layers.

It can also be seen that the curves in the three-point bending vary as a waterfall type with temperature. In the range from room temperature to softening temperature (100 °C), the stiffness and strength of the FMLs significantly reduce with the increment of temperature. When the temperature is near the softening point, deformation is sensitive to both the temperature and the loading rate to a certain extent. As the temperature rises, the bending strength decreases. However, with the increase of loading rate, the stress increases slightly due to strain hardening. The bending strength increases significantly if adopting a high loading rate due to the working hardening of aluminum metal skin. If the temperature is lower than the softening point, deformation is mainly guided by temperature. If temperature is beyond softening point, loading rate is in a leading position of deformation. The bending strength gradually increases with increasing loading rate. Thus, the softening point of resin matrix is the key if a part of FMLs need to be formed by the bending process.

**Figure 12:**
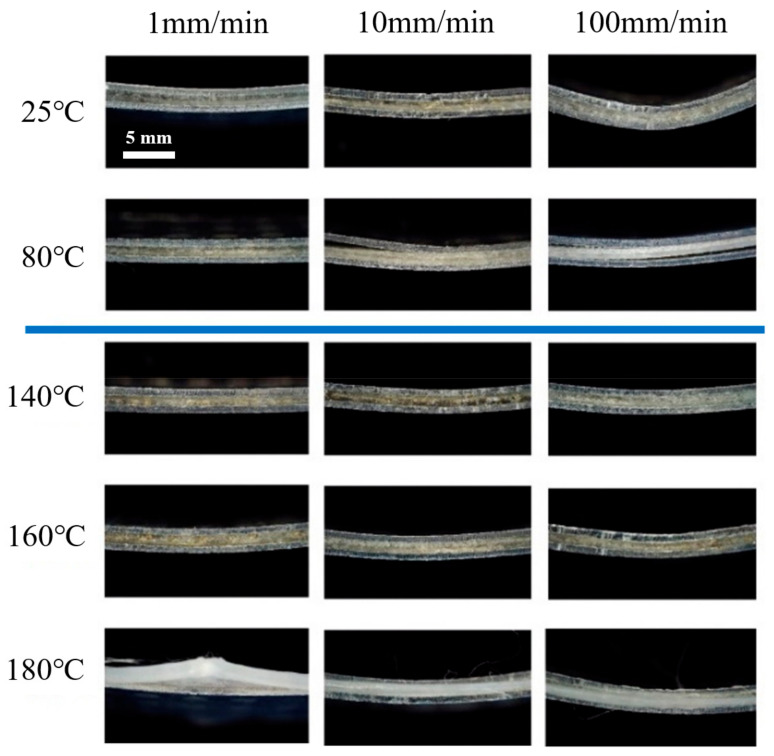
Failure forms under bending tests.

The failure forms were photographed by optical microscope to prove the analysis, as shown in Figure 12, which can reflect the synergistic effect of temperature and loading rate. Longitudinal comparison shows that, when the temperature is lower than the softening point, the softening of polypropylene is not obvious. The failure forms are mainly delamination and crack growth. A higher loading speed led to more distinct delamination. When exceeding the softening point, the plasticity of FMLs was improved. There is no obvious delamination failure. The major failure modes are fiber debonding and sliding. Therefore, the failure modes can be divided into two types: mode I is delamination (below 100 °C; mode II is fiber slipping (at or over 160 °C).

In mode I, the fiber layers always behave elastically, and the metal layers are elastoplastic. The softening point of PP resin (about 100 °C) is characteristic temperature. When the temperature is over the softening point, both glassy state resin and high-elastic state resin exist. For fiber layers, the resin matrix deforms greatly under shear when the resin softens [22]. Meanwhile, two different resin states can cause locally concentrated shear stress gradient, resulting in delamination at the Al/Gf interface. The higher the loading speed, the bigger the stress gradient. It can be confirmed that the softening point of the resin matrix is the characteristic temperature during bend formation.

In mode II, the temperature reaches the melting point of PP resin. The fiber arrangement is changed with the overflow of melted PP resin without bridging effect. The metal layers are still elastoplastic, but the fiber layers cannot bear the external load. The resin matrix is in a viscous state at this time. The fiber can be squeezed under external force, leading to fiber sliding. The failure of FMLs depends on the ductile fracture of metal layers. Therefore, the melt point of PP resin matrix is a characteristic temperature that controls the tensile behavior of Al/Gf/PP laminates.

## 4. Conclusions

In this study, the synergistic effect of temperature and loading rate on the Al/Gf/PP laminate deformation could be concluded. The softening point and the melt temperature of PP matrix are two critical points for deformation. Between the two points, the deformation of the material is controlled by loading rate. Meanwhile, loading rate also controls the deformation gradient. When the resin is in a high-elastic state, the loading rate should be controlled in a relative high range. At the moment, a flat deformation surface emerges. Delamination and matrix cracks did not appear. If the forming temperature is more than the melt temperature of resin, the deformation is hard to control due to resin flow. In a word, the temperature can affect the deformation range as a main factor. Then, the loading rate can control the deformation precisely in the range. Therefore, the Al/Gf/PP laminate should be formed between the softening point and melt point through controlling the loading speed.

## Data Availability

Not applicable.
